# Osteonecrosis of the jaws produced by sunitinib: A systematic review

**DOI:** 10.4317/medoral.22858

**Published:** 2019-05-01

**Authors:** Carmen Vallina, Lucía Ramírez, Jesús Torres, Elisabeth Casañas, Gonzalo Hernández, Rosa-María López-Pintor

**Affiliations:** 1 DDS, Oral Medicine Postgraduate. Department of Dental Clinical Specialties. School of Dentistry. Complutense University, Madrid, Spain; 2 DDS, PhD Student, Oral Medicine Postgraduate. Department of Dental Clinical Specialties. School of Dentistry. Complutense University, Madrid, Spain; 3 DDS, PhD, Professor. Department of Dental Clinical Specialties. School of Dentistry. Complutense University, Madrid, Spain; 4 DDS, PhD, Researcher, Oral Medicine Postgraduate. Department of Dental Clinical Specialties. School of Dentistry. Complutense University, Madrid, Spain; 5 MD, DDS, PhD, Professor, Director Specialty in Oral Medicine. Department of Dental Clinical Specialties. School of Dentistry. Complutense University, Madrid, Spain; 6 DDS, PhD, Associate Professor, Co-director Speciality in Oral Medicine. Department of Dental Clinical Specialties. School of Dentistry. Complutense University, Madrid, Spain

## Abstract

**Background:**

Tyrosine kinase receptor family is involved in tumor growth, pathological angiogenesis and the progression (metastasis) of cancer. Sunitinib (Sutent®) inhibits members of the tyrosine kinase receptor family affecting the induction of angiogenesis and tumor progression. It is not clear if sunitinib increases the risk of osteonecrosis of the jaws (ONJ). The aim of this study was to carry out a systematic review about ONJ related to sunitinib, describing existing cases and possible associated risk factors.

**Material and Methods:**

The PubMed/MEDLINE and Cochrane Library databases were searched without date restriction up to September 2018. We included prospective and retrospective observational studies, cross-sectional studies, clinical cases and series of cases, involving only human subjects. The methodological quality of the studies was assessed using The Joanna Briggs Institute (JBI) and Newcastle-Ottawa tools.

**Results:**

A total of 13 studies fulfilled our inclusion criteria of which 7 were clinical cases, 5 case series and a retrospective study. All the articles were published between 2009 and 2018. Of the 102 patients treated with sunitinib analyzed in this study, 58 developed ONJ, being or having been treated with sunitinib and bisphosphonates or exclusively with sunitinib.

**Conclusions:**

In this systematic review, we found an increase of ONJ in patients who are medicated with other drugs different than bisphosphonates and denosumab. It is necessary that dentists, oral and maxillofacial surgeons as well as oncologists know the risk of ONJ that these antiresorptive drugs could have. There is a need to continue researching in this field with the aim of an increasing knowledge in this area and creating an adequate protocol of action for this population.

** Key words:**Medication-related osteonecrosis of the jaws, osteonecrosis of the jaws, sunitinib, systematic review.

## Introduction

Medication-related osteonecrosis of the jaws (MRONJ) is a chronic osteomyelitis-type of slow and torpid evolution with the presence of one or several bone exposures, without healing for at least 8 weeks, described with relative frequency in patients who have suffered cancer or bone related diseases and have been treated with antiresorptive drugs. According to the current consensus, the clinic and the medical history are enough to establish the diagnosis. There are different risk factors associated with MRONJ. The American Association of Oral and Maxillofacial Surgeons (AAOMS) considers those related to medication (potency of the drug, duration of treatment), local factors (extractions, implants, periodontal surgery, tori, and trauma), demographic and systemic factors (advanced age, tobacco and concomitant treatment with corticosteroids and chemotherapeutic agents) and genetic factors (1).

The first cases of MRONJ that showed an association between bisphosphonates and ONJ date from 2003 (2). So far, ONJ associated with bisphosphonates has been studied in depth. However, the relationship between ONJ with antiresorptive drugs such as RANKL inhibitors (denosumab) and other oncological drugs such as bevacizumab and sunitinib is being studied at the present time (1).

Tyrosine kinase receptor family is involved in tumor growth, pathological angiogenesis and the progression (metastasis) of cancer. Sunitinib (Sutent®) inhibits members of the tyrosine kinase receptor family including platelet-derived growth receptors (PDGFRD and PDGFRE), vascular endothelial growth factors (VEGFR1, VEGFR2 and VEGFR3), stem cell factor receptor (KIT), tyrosine kinase type 3 (FLT3), the colony stimulating factor 1R (CSF-1R) and the neurotrophic factor receptor derived from the glial cell line (RET) (3,4). By inhibition of this signaling pathway, sunitinib affects the induction of angiogenesis and tumor progression. It was introduced for the first time in the US in 2006 in order to treat renal cell carcinoma, as well as gastrointestinal, pulmonary, thyroid and hematological tumors (3,5). Different studies have demonstrated its efficacy, showing remarkable delays in the progression of the disease and a significant benefit in overall survival (6). The first cases of ONJ related to sunitinib date from 2010, when 27 cases of ONJ were described in 100,000 patients treated with this drug, in the period between January and November 2010 in the United Kingdom (7).

However, it is not clear if sunitinib is a causal agent of MRONJ. The cases described are not so numerous as in the case of bisphosphonates. It is believed that sunitinib causes ONJ because it slow down bone remodeling and antagonizes the process of mucosal healing by inhibiting surrounding fibroblasts and endothelial cells, causing bone exposure after dental treatment (8). Other authors suggest that the reduction of angiogenesis caused by sunitinib may impair the host’s defenses against infection, which increases the risk of necrosis (9).

The objective of this work is to carry out a systematic review about ONJ produced by sunitinib, by describing the existing cases and the possible associated risk factors.

## Material and Methods

This systematic review was prepared in accordance with the “Preferred Reporting Items for Systematic Review and Meta-Analysis Protocols” (PRISMA-P, 2015) (10). The addressed focused PECOS (population, exposure, comparison, and outcome) question was as follows: Do patients who have received or are receiving sunitinib as treatment for oncological processes, either exclusively or in combination with other drugs associated to ONJ, suffer from MRONJ?

As a population we consider those patients who suffer from oncological pathologies and receive sunitinib. The exposure refers to the fact these patients have been or are being treated with sunitinib. The risk of ONJ will be compared, whenever possible, between those who have received exclusively sunitinib and patients who have received sunitinib in combination with bisphosphonates or other anti-angiogenic drugs. It will always be determined as a result that the patient has developed ONJ, defined according to current criteria (11).

-Eligibility Criteria

We have included 1) prospective and retrospective observational studies, cross-sectional studies, clinical cases and series of cases, 2) involving only human subjects, 3) without date restriction, 4) with full text availability, 5) written in English and 6) published in scientific journals.

The exclusion criteria were 1) articles about ONJ in which patients were not receiving or had not received treatment with sunitinib, 2) systematic and bibliographic reviews, letters to the editor and experimental studies on animals, 3) only the summary was available and 4) written in a language other than English.

-Information sources and search strategy

**Figure 1:**
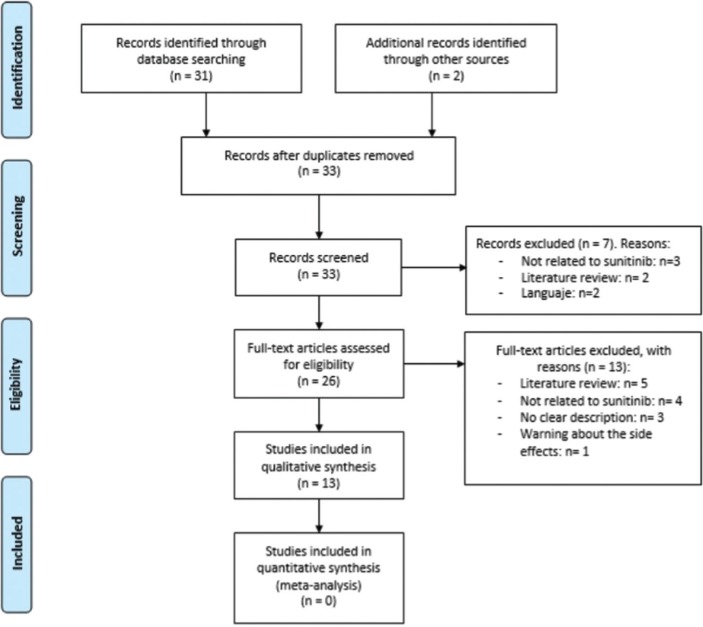
Flow diagram of the literature search.

An exhaustive search of the literature was carried out, without restriction of date until September 24, 2018 in PubMed/MEDLINE and the Cochrane Library electronic databases. The keywords used were “sunitinib” AND “osteonecrosis” OR “osteonecrosis of the jaw” OR “osteonecrosis of the jaws”. Two independent researchers (CV, LR) compared search results to ensure completeness. Duplicates were removed and the full title and abstract of each remaining article were screened individually (Fig. 1). Any differences in the selection of eligible studies were resolved by discussion with a third reviewer (RMLP).

-Data collection.

Two independent reviewers (CV, LR) extracted data from selected articles that met inclusion criteria. Any disagreement was resolved with the help of a third reviewer (RMLP). The following information was collected: 1) General characteristics of the selected studies: title of article, first author, year, type of study, tittle of the journal in which it was published, center and country where it was carried out. 2) Characteristics of the patients studied: number, sex, age, and illness for which they were taking sunitinib. 3) Data regarding sunitinib: dose, duration of treatment and other concomitant treatments. 4) Data related to ONJ: definition, location, stage, triggering factors and treatment for ONJ ( Table 1,  Table 1 continue,  Table 1 continue-1,  Table 1 continue-2).

**Table 1:**
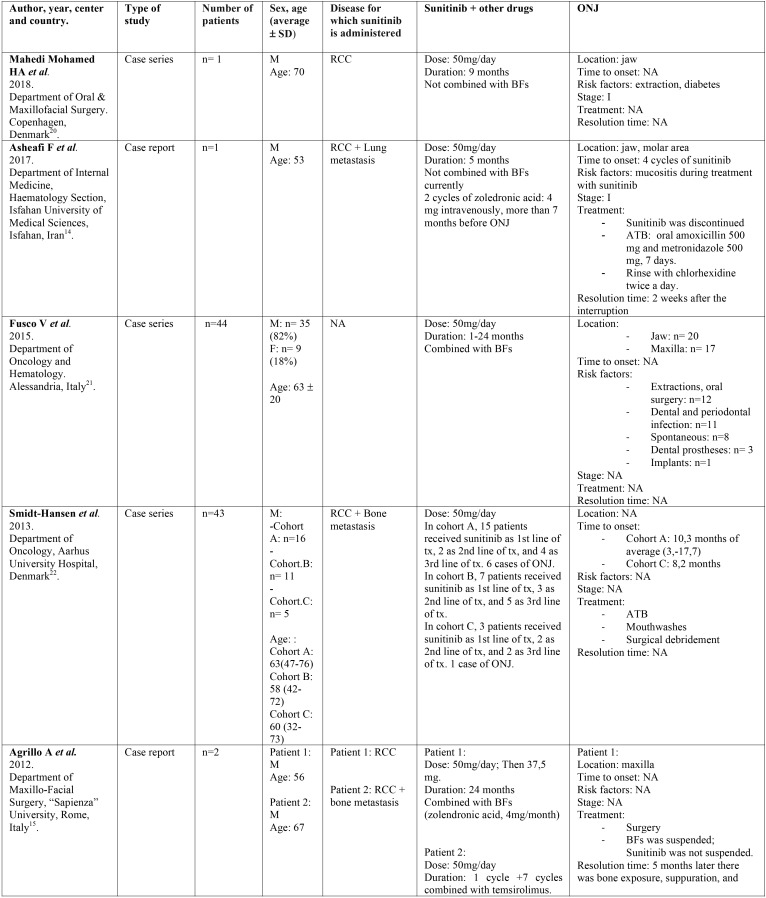
Description of identified studies.

**Table 1 continue:**
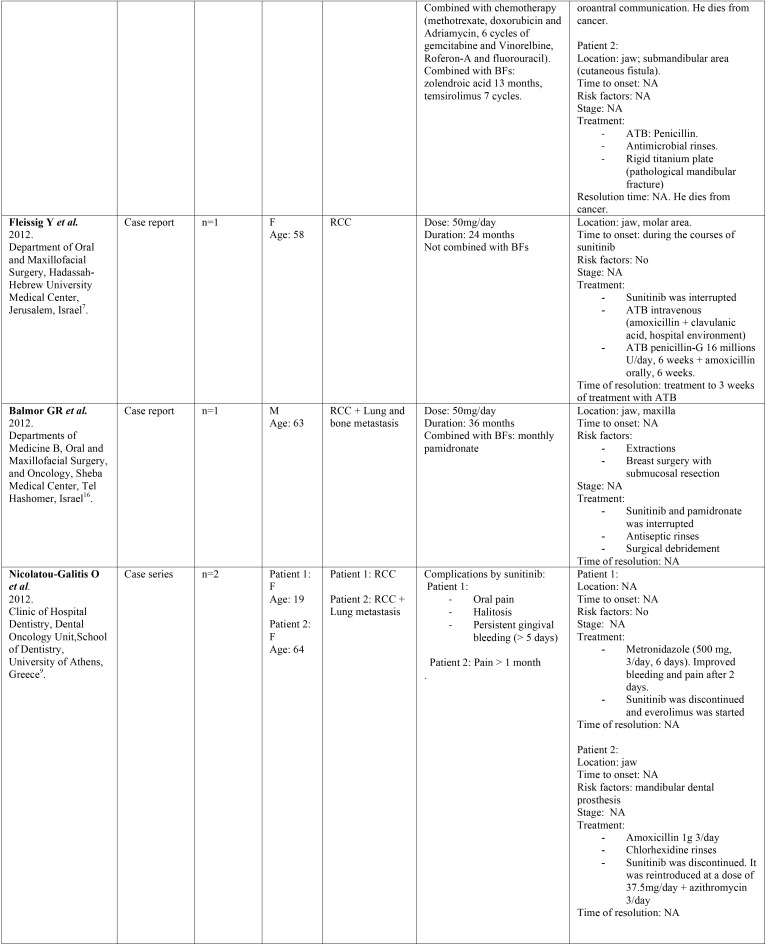
Description of identified studies.

**Table 1 continue-1:**
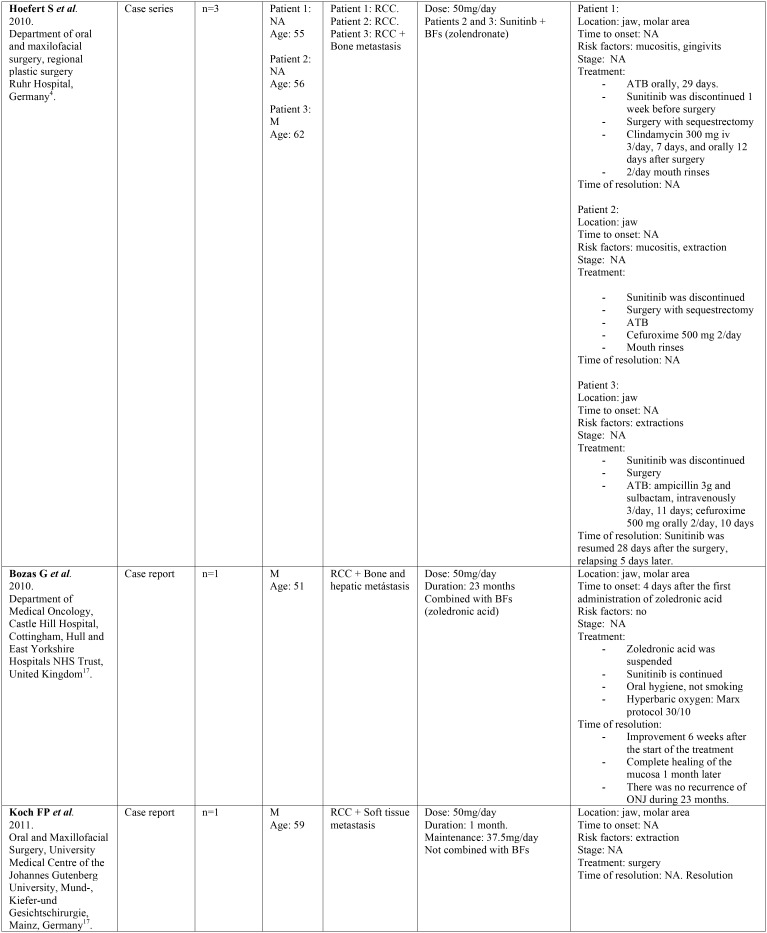
Description of identified studies.

**Table 1 continue-2:**
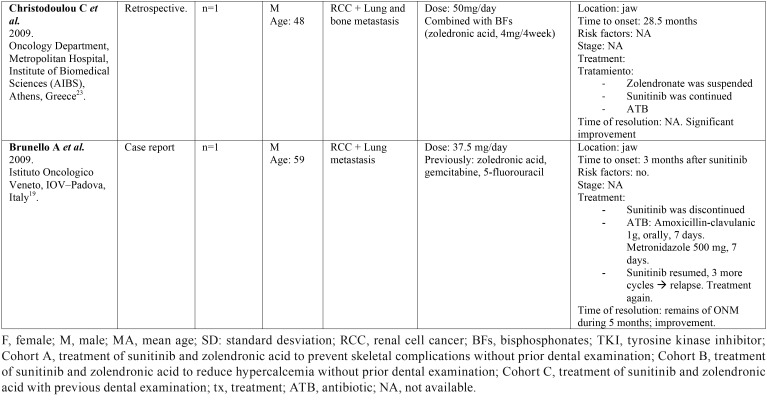
Description of identified studies.

-Risk of bias in individual studies 

Two independent reviewers (CV, LR) evaluated the methodological quality of the eligible studies. If there were no agreement on one paper, it was evaluated by a third reviewer (RMLP). For this evaluation, the critical tools of The Joanna Briggs Institute (JBI) (12) for case series and clinical cases and the Newcastle-Ottawa’s scale (13) for cohort studies were used. We use the JBI clitical appraisal tools for case reports and case series, according to the type of article. The bias is evaluated through a checklist of 8 questions for case report and 10 questions for case series. Each question is specified in  Table 2 and  Table 3 concerning risk of bias. Finally, an overall appraisal is made of each article determining if the risk of bias is low (included), high (excluded), or uncertain (more information needs to be sought). We considered a low risk of bias if the answers “yes” were ≥50%, high risk of bias if the answers “no” were ≥50%, and uncertain risk of bias if the “unclear” answers were ≥50% (12).

**Table 2:**
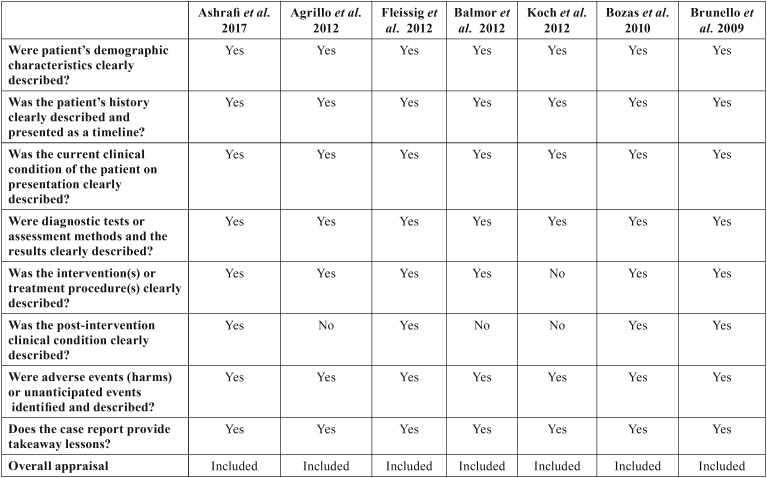
Risk of bias for case reports.

**Table 3:**
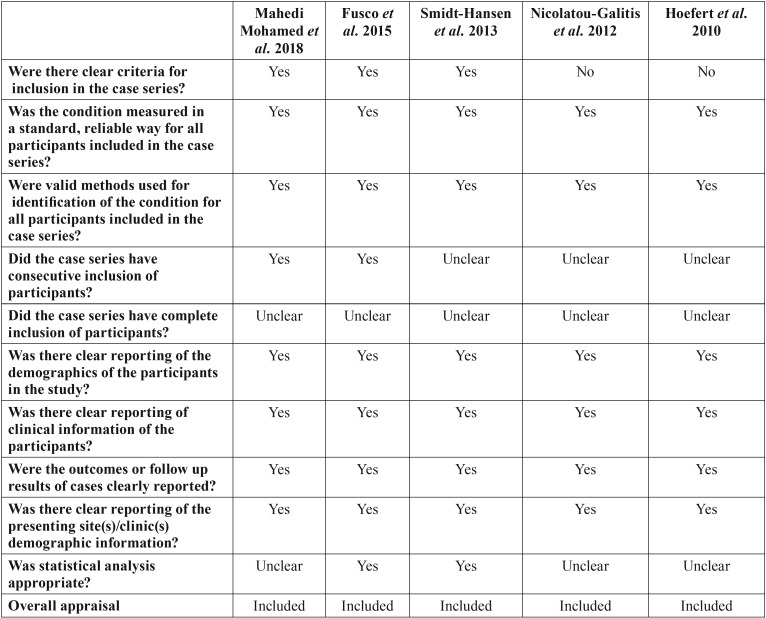
Risk of bias for case series.

We used Newcastle-Ottawa’s scale to assess the risk of bias of the included retrospective study. The bias is evaluated through a questionnaire divided into 3 sections. The first one is “Selection” and it has 4 items. The second section is called “Comparability” and it has only 2 items about the comparability of cohorts on the design or analysis. The last section is “Outcomes” and it has 3 items about the assessment of outcome and follow-up. Each question is specified in  Table 4. We considered a low risk of bias if the study had ≥ 5 of the stars (13). This Newcastle-Ottawa’s scale is shown in T Table 4.

**Table 4:**
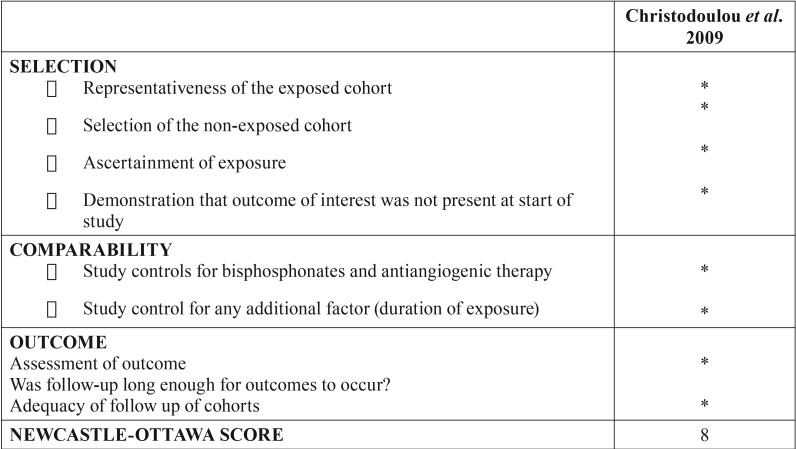
Risk of bias for retrospective studies.

-Risk of bias across studies

**Figure 2:**
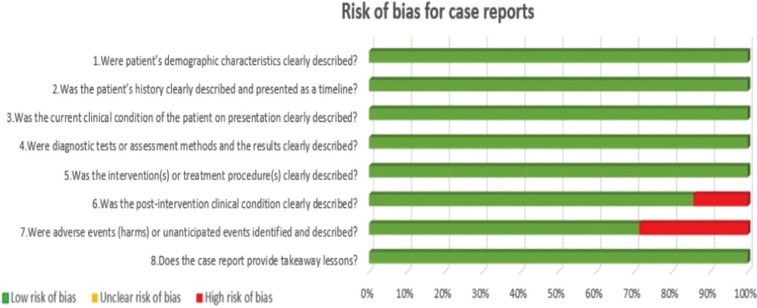
Risk of bias across studies for case reports.

**Figure 3:**
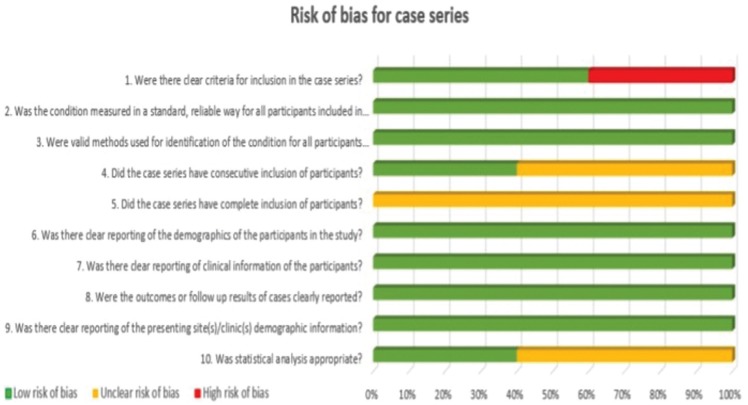
Risk of bias across studies for case series.

Graphs were made to evaluate the risk of bias of all the studies, grouping them according to the type of study. In this way, the risk of bias of the included articles was evaluated jointly, paying attention to the different questions posed by the JBI (Figs. 2,3). No graph was shown for cohort studies because it was only one study.

## Results

-Study selection 

The search strategy yielded 33 results, without duplicates. The two independent researchers (CV, LR) reviewed all the titles and abstracts of the 33 articles. Seven articles were excluded: 2 papers due to language (French, Hungarian), 3 articles for not being related to sunitinib and 2 for being bibliographic reviews. (Annex 1).

After reading the full text, another 13 articles were excluded: 5 for being bibliographic reviews, 4 for not treating sunitinib, 3 for not explicitly describing the medication that patients took (not being able to obtain the number of patients medicated with sunitinib), and one for being a warning about the side effects of bevacizumab and sunitinib (Annex 1). Finally, 13 papers were included in the systematic review (Fig. 1).

-Study characteristics

Of the total number of studies included in this systematic review, we found 7 case reports, 5 case series and a retrospective study. All the articles were published in the last decade, between the years 2009 and 2018. The studies have been carried out in different countries: Italy, United Kingdom, Denmark, Greece, Germany, Israel and Iran. They were published in journals of different medical specialties among which are oral medicine, oral and maxillofacial surgery, oncology, urology, pharmacology and rheumatology. All the studies included a total of 102 patients treated with sunitinib, of which 59 developed MRONJ. The characteristics of the patients studied and the data relative to sunitinib and ONJ are shown in  Table 1.

-Risk of bias of the studies individually

Following the criteria provided by the JBI (12) and Newcastle-Ottawa (13) quality assessment scales, the risk of bias in the studies was assessed. As shown in Table 2, the case reports provided by Ashrafi *et al.* (14), Agrillo *et al.* (15), Fleissig *et al.* (7), Balmor *et al.* (16), Bozas *et al.* (17), Koch *et al.* (18) y Brunello *et al.* (19) have a low risk of bias.

 Table 3 shows the series of cases carried out by Mahedi Mohamed *et al.* (20), Fusco *et al.* (21), Smidt-Hansen *et al.* (22), Nicolatou-Galitis *et al.* (9) and Hoefert *et al.* (4) that presented a low risk of bias.

In the same way, following the criteria provided by the Newcastle-Ottawa´s scale, the retrospective study completed by Christodoulou *et al.* (23) had a low risk of bias, obtaining a good score (8 stars) (T Table 4).

-Results of individual studies ( Table 1)

Of the 102 patients treated with sunitinib analyzed in this study, we can observe that 23 (22.54%) were women and 77 (75.49%) were men. Data from 2 (1.96%) patients were not available (5). Regarding age, the average age was 57 years (minimum-maximum 19-70). Of these patients 58 (56.86%) developed ONJ, of which 38 were males (66%), 11 females (19%) and 9 cases did not specify sex (15%). The average age of the patients with ONJ was 56 years.

Most patients received other medications that alter bone remodeling such as bisphosphonates or denosumab in addition to sunitinib. Forty-nine patients (84%) with ONJ were under concomitant treatment with sunitinib and bisphosphonates, 5 patients (9%) were treated exclusively with sunitinib, one patient (2%) had previously received bisphosphonates although at present he was not treated with them, and another patient (2%) was under combined treatment with sunitinib, bisphosphonates and chemotherapy.

Regarding the local risk factors, the authors highlight extractions and dentoalveolar surgery (n=17, 29%), followed by dental or periodontal infections (n=11, 19%), mucositis due to chemotherapy (n=3, 5%), use of removable dentures (n=3, 5%) or implant insertion (n=1, 2%). In 8 cases the ONJ was spontaneous (14%) and in 15 cases (26%) the associated risk factors were not specified.

Patients received sunitinib orally, and the duration of the treatment ranged from 1 to 36 months. Most of the patients with ONJ received sunitinib at a dose of 50 mg/day (91%), 4% at a dose of 37.5 mg/day and 2% received first 50 mg/day and were subsequently maintained at a dose of 37.5 mg/day. Doses of 2 patients were not available (3%) (9).

Regarding ONJ location, 8 cases (13%) did not specify the location and in the remaining cases 34 occurred in the mandible (54%) and 21 in the maxilla (33%). Only in two cases was the stage specified at the time of diagnosis, being stage I (14,20).

With regards to the treatment, 26% of the subjects with ONJ received antibiotic therapy, chlorhexidine mouthwashes were recommended in 18% of cases and surgery was performed in 16% of cases. The authors proceeded to suspend sunitinib in 18% of the cases, while the bisphosphonates were suspended in 16%. In the 3% of patients sunitinib dose was reduced from 50 mg/day to 37.5 mg/day, and in 3% hyperbaric oxygen therapy was used.

Analyzing the improvement, it was only evaluated in four cases (8,14,17,19). In the first case, improvement was observed 2 weeks after the interruption of sunitinib, and in another case 3 weeks after the interruption of sunitinib. The third patient improved after 6 weeks after stopping the bisphosphonate, while continuing with sunitinib. In the last case, improvement was observed, although with the presence of ONJ remnants, 5 months after the suspension of sunitinib.

-Risk of bias across studies

In Figure 2, it can be observed that in case reports most of the studies have a low risk of bias. Only in the question “Was the post-intervention clinical condition clearly described?” 15% corresponded to a high risk of bias, and in the question “Were adverse events (harm) or incapacitated events identified and described?” 30% had a high risk of bias.

Figure 3 shows the risk of global bias of case series. All the articles have an uncertain risk in the question “Did the case series have complete inclusion of participants?”. Approximately 50% of the articles have had an uncertain risk of bias in the questions “Did the case series have consecutive inclusion of participants?”, and “Was the statistical analysis appropriate?”. In addition, we have considered that 30% of the studies have a high risk of bias in the question “Were there clear criteria for inclusion in the case series?”.

It was not possible to generate a figure for the retrospective studies, because there was only one study with these characteristics. Furthermore, it was not possible perform a meta-analysis due to the quality of the included studies: case series, case reports and only one retrospective study.

## Discussion

-Summary of evidence

The introduction of different antiangiogenic drugs such as sunitinib, in the therapeutic regimen of cancer (22) has caused an increase of ONJ related to other drugs different than bisphosphonates. Most patients receiving sunitinib are related to the treatment of renal cell carcinoma. This type of cancer represents approximately 2% of all cancers worldwide and its incidence of bone metastases is around 30-35% (21,24). For this reason, several drugs such bisphosphonates, denosumab or sunitinib are used for their treatment, mainly to prevent bone destruction and tumor growth (4,9,24). It is estimated that 16% patients with cancer treated with sunitinib, alone or in combination with bisphosphonates, suffer from ONJ (6). For this reason, the European Medicines Agency issued safety warnings about the risk of ONJ during treatment with sunitinib (6,21). Moreover, the AAOMS maintains that ONJ can develop in patients undergoing treatment with antiangiogenic agents such as sunitinib (25). However this association has not been analyzed in depth (20).

In this paper, we have analyzed studies that have shown the presence of ONJ in patients treated with sunitinib, alone or combined with bisphosphonates. Most authors agree that the risk of ONJ is greater when antiangiogenic drugs are combined with bisphosphonates (3,8,14,16,21,23), others such as Ashrafi *et al.* maintain that ONJ is more related to the use of sunitinib than to bisphosphonates, because the side effects improve with the disruption of sunitinib (14). On the other hand some authors argue that there is not enough evidence in the literature about the cause-effect relationship between sunitinib and ONJ (26).

-ONJ in patients receiving concomitant treatment with sunitinib and bisphosphonates

As we have seen, in this revision 49 from 58 (84%) patients with ONJ were under concomitant treatment with sunitinib and bisphosphonates. The retrospective study by Beuselinck *et al.* shows that the incidence of ONJ in patients with renal cell cancer increased up to 10% when combined treatment with bisphosphonates and sunitinib were added (21). Some authors justify this higher risk due to the greater cumulative toxicity profile in patients treated with bisphosphonates and sunitinib, since both drugs have an antiangiogenic effect (9). However, it is remarkable that ONJ was only observed in patients treated with zoledronate in combination with sunitinib and not with the combination of zoledronate with sorafenib, pazopanib, temsirolimus or immunotherapy based on IL-2 (22,25). This could be explained because sunitinib is between 10%-30% more potent against VEGFR and PDGF than the other drugs (8,16,18,21). Another reason is that this drug can also cause damage to the oral mucosa producing gingival inflammation and mucositis, delaying wound healing and favoring infection. This in turn would increase the risk of ONJ (9,14). Indeed, some authors describe cases in which oral mucositis preceded bone exposure (4).

-ONJ local risk factors associated with sunitinib

It has been suggested that the local risk factors of ONJ associated with sunitinib are similar to those of ONJ associated with bisphosphonates. Among the dental risk factors, it should be noted that tooth extraction is the most frequent triggering event (27). But there are other precipitating local factors such as other types of dentoalveolar surgery, the continued trauma to the mucosa (such as that produced by a removable prosthesis), the presence of exostosis, oral infection, the presence of periodontitis and poor oral hygiene (4,9,20,25). There are authors who also consider tobacco as a risk factor for ONJ (17).

In our work, we have observed how the local risk factors most frequently associated with the onset of ONJ were tooth extraction and dentoalveolar surgery, followed by dental or periodontal infections. Other risk factors were mucositis due to chemotherapy, the use of removable dentures or implant insertion. We found that 14% of cases were spontaneous.

-ONJ systemic risk factors associated with sunitinib

There are also a number of systemic risk factors for ONJ, including the duration of administration of antiresorptive drugs, the dose, the simultaneous use of antiangiogenic and antiresorptive drugs, treatment with chemotherapy or concomitant corticosteroids, or diseases such as diabetes (16,27). Since the introduction of targeted therapies, the life expectancy of patients with metastatic renal cell cancer has tripled, but this also leads to prolonged exposure to treatments such as bisphosphonates and anti-angiogenic drugs that increase the risk of ONJ (9,21). Also chemotherapy can be another systemic risk factor. There are controversies as to whether sunitinib is a conventional chemotherapy treatment or not. Authors such as Hoefert *et al.* differentiate sunitinib from conventional chemotherapy according to the treatment length. Conventional chemotherapy is given for a defined period of time, but sunitinib can be maintained for a longer period of time, sometimes even years, which also increases the risk of ONJ (4,14). There are articles included in this study that show how the time elapsed between the initiation of treatment with sunitinib and the appearance of ONJ varied from 7 to 9 months (4). In comparison with bevacizumab, Mahedi Mohamed *et al.* suggest that the duration of treatment with sunitinib before the onset of ONJ is longer (20).

All patients included in this study received sunitinib for renal cell carcinoma, with or without metastasis. The patients received this drug orally, and the duration of the treatment ranged from 1 to 36 months. Ninety-one per cent of the patients with ONJ received sunitinib at a dose of 50 mg/day. Of the cases of ONJ, only 5 patients (9%) received sunitinib without being associated with other medications that cause ONJ. As we have seen 49 patients (84%) were administered sunitinib in combination with bisphosphonates simultaneously, with zolendronic acid predominating.

-Location of ONJ with sunitinib treatment

In previous studies performed on the ONJ in patients medicated with bisphosphonates it was observed that the lesions occurred mostly in the mandible (68.1%), and to a lesser extent in the maxilla (27.7%) or in both jaws (4.2%) (27). Regarding the ONJ lesions of this study, 54% of cases occurred in the mandible and 33% in the maxilla. So it seems that the location is similar to the case of bisphosphonates.

-Treatment protocol

The established protocol in a case of ONJ associated with sunitinib is very variable and there are discrepancies among the authors. There are authors who propose that administration of systemic antibiotics, exhaustive oral hygiene, the use of rinses with chlorhexidine, together with the temporary interruption of sunitinib can lead to clinical improvement of the oral lesions (9,14,16,19). The healing time of the soft tissues after the interruption of sunitinib seems to be relatively short, in some studies it is estimated between 2-4 weeks (4,9), however when treatment is restarted ONJ relapses again (4,19).

Other authors such as Hoefert *et al.* (4) suggest that since most of the side effects are reversible, treatment with sunitinib should not be stopped, although dose adjustment has been recommended. Agrillo *et al.* (15) also are in favor of continuing sunitinib, although recommends bisphosphonate disruption in patients under combining treatment.

If we observe the included studies of this systematic review, the most frequent treatments of ONJ were antibiotic therapy (26%) (4,8,9,14,15,19,22,23), chlorhexidine mouthwashes (18%) (4,9,14,15,16,22), sunitinib (18%) (4,8,9,14,15,16,19), and bisphosphonates suspension (16%) (15,16,17,23), and surgery (16%) (4,15,16,18,22). The improvement was only evaluated in four cases (7,14,15,19). In 3 of these cases improvement was observed after interruption of sunitinib, but the improvement time was very variable, from 2 weeks to 5 months (7,14,19). Interestingly the fourth patient improved following 6 weeks after stopping the bisphosphonate, while continuing with sunitinib (17).

Prevention is the main objective of disease control (16), however there are no specific guidelines nowadays (4). Some authors such as Nicolatou-Galitis *et al.* advise using the same recommendations used in patients receiving bisphosphonates (9). The oral examination prior to treatment with sunitinib is aimed at reducing the oral microbial load and infection; this will reduce the degree of oral mucositis induced by chemotherapy, which may reduce the risk of developing MRONJ (9). In a study of patients with renal cell carcinoma and bone metastases treated with zoledronic acid combining with target therapy carried out by McKay *et al.* (24), the ONJ rate was 29% and was only observed in patients treated with sunitinib. After the introduction of mandatory oral examinations before the use of these drugs, the rates decreased to 11% (24). This theoretically justifies the dental check-ups prior to treatment with sunitinib and other antiresorptive drugs. It is also important to perform periodic check-ups during treatment because oral changes, different to ONJ, appear in 10-50% of patients treated with sunitinib and include dysgeusia, xerostomia, mucosal sensitivity, oral pain, gingival bleeding, gingivitis necrotizing and oral mucositis (4,9).

-Limitations

Regarding the limitations of this systematic review, we could highlight the type and characteristics of most of the included studies, since case series or isolated clinical cases with very low methodological quality predominate. In addition, each author defined different characteristics of their patients, making it difficult to unify the aspects that may influence the development of ONJ. Therefore, no meta-analysis was performed.

For this reason, there is a need to better evaluate the incidence of ONJ in large cohorts of patients prospectively, given the increasing use of treatment with antiresorptive drugs, targeted therapies, antiangiogenic drugs and bisphosphonates in the modern era of oncology.

## Conclusions

With the introduction of sunitinib as a new treatment, a growing number of patients with ONJ have been reported, it is suggested that the antiangiogenic activity of the targeted agents can inhibit bone remodeling, delay the healing of the tissues and thus enhancing the development of ONJ. In addition, the mucositis produced by this drug may also increase the risk of ONJ. Most authors agree on an increased risk of developing ONJ in those cases in which bisphosphonates and sunitinib are combined.

To reduce the incidence of ONJ, a clinical examination should be carried out on all patients before and periodically during treatment with these drugs. A correct oral situation and careful oral hygiene play a fundamental role in preventing this pathology.

It is necessary that dentists, oral and maxillofacial surgeons as well as oncologists know this relationship, in order to establish a protocol of periodic oral check-ups to reduce the risk of ONJ and make an early diagnosis.

Knowledge about the relationship between ONJ and new target therapies such as sunitinib is still limited. There are discrepancies in the protocol to be followed in case of ONJ, although it seems that the suspension of the drug improves the situation. For this reason, there is an imperative need to continue researching in this field.
